# Impact of Remote Titration Combined With Telemonitoring on the Optimization of Guideline-Directed Medical Therapy for Patients With Heart Failure: Protocol for a Randomized Controlled Trial

**DOI:** 10.2196/19705

**Published:** 2020-10-13

**Authors:** Veronica Artanian, Valeria E Rac, Heather J Ross, Emily Seto

**Affiliations:** 1 Institute of Health Policy, Management and Evaluation Dalla Lana School of Public Health University of Toronto Toronto, ON Canada; 2 Ted Rogers Centre for Heart Research Peter Munk Cardiac Centre University Health Network Toronto, ON Canada; 3 Toronto Health Economics and Technology Assessment (THETA) Collaborative University Health Network Toronto, ON Canada; 4 Toronto General Hospital Research Institute University Health Network Toronto, ON Canada; 5 Centre for Global eHealth Innovation Techna Institute University Health Network Toronto, ON Canada; 6 Department of Medicine University of Toronto Toronto, ON Canada

**Keywords:** telemonitoring, telemedicine, remote titration, mHealth, heart failure

## Abstract

**Background:**

Guideline-directed medical therapy (GDMT), optimized to maximum tolerated doses, has been shown to improve clinical outcomes in patients with heart failure (HF). Timely use and optimization of GDMT can improve HF symptoms, reduce the burden of hospitalization, and increase survival rates, whereas GDMT deferral may worsen the progression of HF, decrease survival rates, and predispose patients to poor outcomes. However, studies indicate that GDMT remains underused, with less than 25% of patients receiving target doses in clinical practice. Telemonitoring is a potential component in the management of HF that can provide reliable and real-time physiological data for clinical decision support and facilitate remote titration of medication.

**Objective:**

The primary objective of this study is to evaluate the impact of remote titration facilitated by telemonitoring on health care outcomes, with a primary outcome measure being the proportion of patients achieving target doses. The secondary objective is to identify the barriers and facilitators that can affect the implementation and effectiveness of the intervention.

**Methods:**

A mixed methods study of a smartphone-based telemonitoring system is being conducted at the Peter Munk Cardiac Centre (PMCC), University Health Network, Toronto. The study is based on an effectiveness-implementation hybrid design and incorporates process evaluations alongside the assessment of clinical outcomes. The effectiveness research component is assessed by a two-arm randomized controlled trial (RCT) aiming to enroll 108 patients. The RCT compares a remote titration strategy that uses data from a smartphone-based telemonitoring system with a standard titration program consisting of in-office visits. The implementation research component consists of a qualitative study based on semistructured interviews with a purposive sample of clinicians and patients.

**Results:**

Patient recruitment began in January 2019 at PMCC, with a total of 76 participants recruited by February 24, 2020 (39 in the intervention group and 37 in the control group). The final analysis is expected to be completed by the winter of 2021.

**Conclusions:**

This study will be among the first to provide evidence on the implementation of remote titration facilitated by telemonitoring and its impact on patient health outcomes. The successful use of telemonitoring for this purpose has the potential to alter the existing approach to titration of HF medication and support the development of a care delivery model that combines clinic visits with virtual follow-ups.

**Trial Registration:**

ClinicalTrials.gov NCT04205513; https://clinicaltrials.gov/ct2/show/NCT04205513

**International Registered Report Identifier (IRRID):**

DERR1-10.2196/19705

## Introduction

### Background

Heart failure (HF) is a global public health problem affecting an estimated 26 million worldwide [1]. Researchers estimate that more than 1 million people in Canada are living with HF, about 50,000 new cases are diagnosed each year, and HF costs the Canadian health care system Can $2.8 (US $2.12) billion annually [2,3]. The rapidly aging population in developed countries and improved prognosis of HF are contributing to the increasing prevalence of people with HF. However, although HF prognosis has improved, the long-term mortality rates for the condition remain high. Approximately 1 in 3 patients admitted to the hospital with HF still die within a year and approximately 1 in 2 die within 5 years [4].

In addition, HF accounts for 1% to 2% of direct health care expenditure in developed countries [5] and the cost to our health care system is expected to grow with the aging of the population and rising prevalence of HF. In Canada alone, studies found that in 2013, HF hospitalizations accounted for Can $482 (US $364.34) million in spending. By 2030, the amount is estimated to increase to Can $722 (US $545.75) million [6].

Over the past few years, significant progress has been made in pharmacological therapies for HF. Guideline-directed medical therapy (GDMT) for patients with HF, comprising angiotensin-converting enzyme inhibitors (ACEIs), angiotensin receptor blockers (ARBs), angiotensin receptor-neprilysin inhibitors (ARNIs), beta blockers (BBLs), and mineralocorticoid receptor antagonists (MRAs), has been shown through randomized controlled trials (RCTs) to improve symptoms, reduce the burden of hospitalization, and increase survival rates [7]. Conversely, a meta-analysis conducted in 2017 by Zaman et al [8] showed that a 1-year deferral of treatment could reduce the 1-year survival rate from 90% (if treated) to 78%. GDMT deferral may also worsen HF progression and predispose patients to worse outcomes [9]. Clinical guidelines recommend up-titrating these treatments to maximum tolerated doses [10]. However, these successes have not fully translated into clinical practice, as studies and registries consistently report that evidence-based pharmacotherapies for HF are severely underutilized [11,12].

Barriers including patient-related factors such as time constraints and financial limitations, physician-related issues such as knowledge of drug therapy optimization, and institution-related logistical issues surrounding clinic visits often complicate the titration process [13]. In addition, the dynamic nature of HF presents challenges in patient care and management, with patients receiving care in primary, acute, and community care settings and with frequent transitions between care providers [14]. These frequent transitions of care across multiple providers and multiple settings are poorly coordinated and often exacerbate the difficulty of medication titration [15]. All these factors impede timely optimization of vital therapy for patients with HF, which is particularly detrimental because delays in therapy can lead to significant disease progression that may have been preventable [9].

Telemonitoring is a potential component in the management of HF that can provide reliable and real-time physiological data for clinical decision support, alerting, and patient self-management. Telemonitoring enables patients to track vital signs and symptoms and receive automated instruction and clinical intervention during *teachable moments* (ie, clear actions are provided when the context is most appropriate). The acquired physiological and symptom data can also help inform clinical decisions by health care providers, such as remote titration of medication [16]. 

### Objectives

This study aims to explore how the combination of remote titration and telemonitoring affects GDMT optimization compared with standard of care. The study takes a dual focus in assessing both the clinical effectiveness and implementation of the intervention. The primary objective of this study is to evaluate the impact of remote titration facilitated by telemonitoring on health care outcomes, including the proportion of patients achieving target doses, time to dose optimization, and patient health outcomes. The secondary objective of this study is to obtain a deeper understanding of the experiences of clinicians and patients with HF participating in the remote titration program to identify factors that affect the implementation of the intervention.

## Methods

### Study Design Overview

The study is based on a type 1 effectiveness-implementation hybrid design. In this type of study, the primary aim is to determine effectiveness, and the secondary aim is to explore the implementation of the innovation [17]. This is a mixed methods study with 108 patients with HF, consisting of an RCT and a qualitative study. The RCT predominantly addresses the effectiveness component while providing supporting data for the implementation component of the research. The qualitative study predominantly addresses the implementation component while providing supporting data for the effectiveness component. In addition, the study included an internal pilot [18] that aimed to identify the most suitable primary outcome measure and obtain more accurate data to inform an appropriate sample size calculation.

This study has received approval from the research ethics boards of the University of Toronto (research ethics board number 00036655) and the research ethics boards at the University Health Network (UHN; research ethics board number 18-5351), where patients are recruited and patient data are stored. The study has also been registered at ClinicalTrials.gov (NCT04205513).

### Sample Size Calculation

One of the main outcome measures of this study, the number of visits required to complete titration, was used to calculate the initial sample size based on data obtained from the existing literature. Assuming biweekly titration over a period of 3 to 6 months (ie, 9±3 visits in total for the control group) as recommended by HF guidelines [10,19], a reduction of at least 35% in the number of visits for the intervention group, 80% power, and α=.05 (two-sided), the sample size per group was calculated to be 16. Furthermore, assuming that as much as 30% of the patients may be lost to follow-up or cannot be titrated, the sample size per group became 21. Hence, 42 patients were enrolled for the internal pilot portion of the study.

Data from the internal pilot and the literature were combined to perform the final sample size calculation. It was determined that the initially selected primary outcome measure of the number of visits required to achieve titration was not appropriate because clinic visits were strongly affected by external factors unrelated to the intervention. Many patients in the control group attended very sporadic clinic visits, which resulted in a slow and unpredictable titration process. Therefore, the new calculation was based on an alternative primary outcome measure, which was the proportion of patients achieving target doses. In the pilot cohort, 18 of 21 patients (86%) in the intervention group and 10 of 21 patients (48%) in the control group completed titration within 6 months of enrollment. According to an expert panel conducted by the Canadian Cardiovascular Society, most physicians (55%) believed that the entire triple therapy titration to maximum tolerated or target doses should be completed within 4 months, 93% believed that this should be done within 6 months, and all respondents agreed that every titration would not necessarily require a face-to-face visit [19]. The titration completion rates reported in the literature vary, and the timelines are not always clear. However, multiple studies reported that 17% to 43% of patients achieve target doses within 3 to 6 months [12,20,21].

In light of the varying ranges provided by available data and to ensure that the sample size of the full study would be sufficient to detect statistical significance, a higher completion rate among those available was used for the control group, and a slightly more conservative completion rate was used for the intervention group. Therefore, rates of 45% and 75% were used for the control and intervention groups, respectively. The calculation assumed a power of 80%. An alpha of .025 (two-sided) was used, instead of .05, to account for the single interim analysis conducted. On the basis of this, the sample size per group was calculated to be 49. Furthermore, assuming that up to 10% of the patients may be lost to follow-up or cannot be titrated (based on the observed attrition rate in the internal pilot), the sample size per group was calculated to be 54. Hence, the overall sample size of the study is 108 patients.

### Medly Telemonitoring System

Medly, a mobile phone–based telemonitoring program for patients with HF was launched at UHN in 2016. This program is integrated into the Ted Rogers Centre of Excellence in Heart Function at the Peter Munk Cardiac Centre (PMCC) as part of the standard of care.

Medly enables patients with HF to take clinically relevant physiological measurements with wireless home medical devices in addition to answering symptom questions through the mobile phone app. The measurements are automatically and wirelessly transmitted to the mobile phone and then to a data server. Specifically, patients monitor daily weight, blood pressure, heart rate, and symptoms, and some patients monitor their activity as determined by their cardiologist. Daily reports are typically completed every morning (patients receive an automated reminder call if the measurements are not performed by 10 AM), and patients are instructed to record their blood pressure and symptoms if they feel unwell. Automated self-care instructions that have been carefully developed with health care specialists are sent to the patient in accordance with a rule-based algorithm that analyzes their measurements and reported symptoms [22]. If there are signs indicating deterioration in their status, an email alert is sent to a clinician at the Heart Function Clinic. Clinicians can also view alerts and the patient’s telemonitoring data through a secure web portal. The data are monitored by a dedicated Medly nurse coordinator during working hours and an assigned clinician after hours and on weekends. Medly has a demonstrated positive impact on patient outcomes and patient experience. An RCT conducted with Medly at the Heart Function Clinic and an evaluation of the Medly Program as part of the standard of care found improvements in patient health outcomes and high patient and health care provider satisfaction. Adherence to daily monitoring was high, and the cardiologists and nurse practitioners indicated that Medly improved information transfer from their patients because they received real-time patient information and alerts that supported clinical decision making [23,24].

Therefore, Medly was chosen as the system to support the titration of HF medication for this study. Specifically, the intent was for Medly to be used to provide frequent and real-time data to support clinical decisions on the optimal modification of patients’ medications remotely.

### Study Protocol

#### Participant Enrollment and Randomization

##### RCT

Study participants are recruited from the PMCC Heart Function Clinic. Eligible participants are first identified by the cardiologist. During their usual visit to the Heart Function Clinic, all patients who meet the study’s inclusion and exclusion criteria (listed in Textboxes 1 and 2, respectively), are told about the study and asked by a member of their circle of care if they are willing to speak to the nurse coordinator regarding participation. Patients who agree meet with the coordinator immediately after their visit with the cardiologist, and a written informed consent is obtained from each patient. Patients are then randomized 1:1 into control and intervention groups. A web-based computer-generated randomization tool is used to perform block randomization in blocks of 4. The generated sequence is used to create randomization envelopes, and the nurse coordinator is provided with randomly generated treatment allocations within sealed opaque envelopes. Following enrollment, the envelopes are used to determine if the patient is in the intervention or control group. Cardiologists are notified into which group their patients are randomized.

**Patient inclusion criteria.**

- Able to provide informed consent to participate in the program
- 18 years or older
- Diagnosed as having heart failure (HF) and followed up by a cardiologist at the Peter Munk Cardiac Centre Heart Function Clinic, who has the primary responsibility for management of the patient’s HF
- New York Heart Association Classes I to III
- Stable HF defined as no hospitalization within 1 month
- Patient is not yet at target doses of guideline-directed medical therapy (angiotensin-converting-enzyme inhibitor, and/or angiotensin receptor blocker, and/or beta blocker, and/or angiotensin receptor-neprilysin inhibitors, and/or mineralocorticoid receptor antagonist at suboptimal doses), and hence qualifies for up-titration
- Patient or their informal caregiver speaks and reads English adequately to participate in the program and understand the alerts or prompts in the Medly application
- Ability to comply with using Medly (eg, able to stand on the weight scale, able to answer symptom questions)

**Patient exclusion criteria.**

- Active acutely decompensated heart failure
- Already on target doses of guideline-directed medical therapy
- Inability to titrate medications due to adverse events including:
  - History of angioedema
  - Uncontrolled hypertension
  - Hypotension preventing up-titration
  - Heart rate at rest <56 beats per minute
- Congenital heart disease
- Previous heart transplant or currently awaiting heart transplant
- Acute coronary syndrome; stroke; transient ischemic attack; cardiac, carotid, or other major cardiovascular surgery; percutaneous coronary intervention; or carotid angioplasty within 6 weeks before randomization
- Obstructive or restrictive cardiomyopathy
- Second- or third-degree atrioventricular block without a pacemaker
- Presence of hemodynamically significant mitral and/or aortic valve disease, except mitral regurgitation
- Presence of other hemodynamically significant obstructive lesions of the left ventricular outflow tract, including aortic and subaortic stenosis, which are not controlled with suitable treatment
- Evidence of hepatic impairment defined as alanine aminotransferase or aspartate aminotransferase value >3-fold the upper normal limit. Estimated glomerular filtration rate (eGFR) <30 mL/min/1.73 m^2^ at randomization or >35% decline in eGFR between visits
- Known stenosis of both renal arteries
- Hyper- or hypothyroidism not controlled by treatment
- Hyperkalemia >5.5 mmol/L at randomization
- Hyponatremia <130 mmol/L at randomization
- History of severe asthma or pulmonary disease
- Presence of any other disease, which in the clinician’s opinion would exclude the patient from the study or with a life expectancy of <1 year

##### Qualitative Study

Patients randomized into the intervention group will be invited to participate in individual interviews intended to assess their experience and perception of the program upon titration completion. Maximum variation sampling [25] will be used to interview a varied selection of people. Participants will be purposively selected to represent a range of experiences with the intervention and include men and women, old and young, and patients who reside at varying distances from the clinic.

All health care providers and study staff from the Heart Function Clinic who participate in the remote titration program during the RCT will also be invited to participate in semistructured interviews through an email. Written informed consent will be obtained before the start of any interview.

Semistructured interview guides will be developed to explore the participants’ views on various aspects of the remote titration program. To ensure that the information generated is based on the participants’ unique perspectives, questions will not follow any specific constructs. During the interview, participants will be asked open-ended questions to ascertain their comfort with the intervention and its delivery, any concerns or difficulties they may have had with respect to the intervention, and whether it met their goals or expectations. Follow-up questions will explore topics raised by participants.

#### Intervention Versus Control Groups

The recommended therapeutic approach for patients with HF and reduced left ventricular ejection fraction (LVEF<40%) consists of triple therapy with either an ACEI or ARB or ARNI, BBL, and MRA, titrated to target doses [10]. However, these doses are not often achieved in clinical practice [7]. Patients identified as receiving suboptimal doses of HF medications are enrolled and randomized at a ratio of 1:1 to 1 of 2 treatment groups:

- Control group—standard titration management strategy consisting of regular in-office visits.
- Intervention group—remote titration management strategy consisting of telephone contacts facilitated by data from the Medly system and in-office visits as deemed necessary by the patient’s care team.

Both groups are titrated in accordance with the recommendations of the HF management guidelines. Participants in the intervention group, as per Medly standard of care, are asked to take daily weight and blood pressure readings and answer questions on symptoms. Patients are also provided with requisitions for blood work to be performed at local laboratories when requested. Titration checkpoints are scheduled biweekly unless specified otherwise by cardiologists. Patients are contacted by phone, and medication changes are performed during these calls based on data obtained through Medly and the latest blood work. Patients in the intervention group still visit the hospital for follow-ups at their cardiologist’s discretion and to perform echocardiograms, electrocardiograms (ECGs), and cardiopulmonary exercise testing (CPET).

Participants in the control group attend regular visits and are provided with the current standard of care. As the Medly system is integrated into the PMCC Heart Function Clinic as part of the standard of care, patients in the control group are also monitored through Medly. However, no remote titrations occur, and medication changes are performed during clinic visits. At the clinic visit, patients have their measurements taken, such as their blood pressure, weight, and heart rate. The clinicians inquire about their symptoms, diet, exercise, and adherence to medication. Blood tests, echocardiograms, ECGs, and CPETs are performed as required. Medication changes are performed based on the data collected through these assessments.

The titration process is terminated when patients reach target doses or maximum tolerated doses. A follow-up clinic appointment is scheduled within 3 months of titration completion, as per the standard of care. Throughout this process, the importance of patients’ adherence to program requirements in terms of daily measurements and symptom reporting is emphasized and strictly monitored to ensure prompt identification of potential changes in their condition.

#### Data Collection

##### RCT

The primary outcome measures will be the proportion of patients who achieve target doses and the time to dose optimization. Additional measures will include patient health outcomes (including, but not limited to, New York Heart Association [NYHA] class, LVEF, and brain natriuretic peptide [BNP] levels), the number of visits and/or calls required to achieve target doses, and health care resource utilization.

Information will be obtained by reviewing the patients’ electronic patient record (EPR) charts and Medly data and documentation throughout the study by the study coordinator. Baseline and poststudy medications and dosages for each patient and baseline and poststudy clinical measures, including NYHA class, LVEF, and BNP levels, will be determined through manual EPR chart reviews. Health care utilization will primarily be determined through EPR chart reviews. However, this information will be supplemented through patient self-reporting to account for situations in which patients use services outside of UHN. Data regarding the titration process, such as the number of visits and/or phone calls performed, actions undertaken, and any adverse events that occurred throughout the study, will be documented by the study coordinator. The number of visits, phone calls, and total contact points will be recorded in detail for each group to determine the impact of remote titration on the GDMT optimization process.

##### Qualitative Study

The qualitative study will aim to identify the barriers to and facilitators for implementation. Semistructured one-on-one interviews will be conducted with participants to explore their views on various aspects of the remote titration program. Patients randomized into the intervention group will be interviewed in a quiet and private space within the clinic or over the telephone. Interviews are expected to last between 15 and 30 min. Additional interviews will be conducted until data saturation is reached and the interviewer determines that no new pertinent information is being collected. Health care providers from the Heart Function Clinic who participate in the remote titration program will also be interviewed. The individual interviews are expected to last between 20 and 45 min and will be conducted in the clinician’s office or over the telephone. All interviews will be audio-recorded and transcribed for subsequent analysis.

The schedule for data acquisition is shown in Table 1.

**Table 1:** Schedule for data acquisition indicated by checkmarks at the specified time point.

Data collected		Baseline	Interim analysis	Titration completion	3-month follow-up
Demographics		✓	N/A^a^	N/A	N/A
**Health service utilization**					
	Number of HF^b^-related hospitalizations since enrollment	N/A	✓	✓	✓
	Number of days in the hospital since enrollment	N/A	✓	✓	✓
	Number of emergency department visits since enrollment	N/A	✓	✓	✓
	Number of clinic visits or phone calls since enrollment	N/A	✓	✓	✓
**Clinical outcomes**					
	BNP^c^ levels	✓	✓	✓	✓
	NYHA^d^ class	✓	✓	✓	✓
	LVEF^e^ (%)	✓	N/A	N/A	✓
**Qualitative data**					
	Clinician interviews	N/A	✓	✓	N/A
	Patient interviews	N/A	✓	N/A	N/A

^a^N/A: not applicable.

^b^HF: heart failure.

^c^BNP: brain natriuretic peptide.

^d^NYHA: New York Heart Association.

^e^LVEF: left ventricular ejection fraction.

#### Data Analysis

##### RCT

Descriptive, parametric, and nonparametric statistics will be performed. Statistical analyses will be selected in accordance with the data under review and the required level of comparison: McNemar tests will be performed on binary baseline and poststudy data, whereas chi-square tests will be performed to compare binary poststudy data between the intervention group and the control group; paired Student *t* tests and Wilcoxon signed-rank tests will be performed on baseline and poststudy data for normally and not normally distributed data, respectively. Independent Student *t* tests and Mann-Whitney tests will be performed to compare poststudy data between the intervention group and the control group for normally and not normally distributed data, respectively.

##### Qualitative Study

Conventional content analysis [26] will be used to analyze the transcribed interviews, and coding will be performed using NVivo software (QSR International). A conventional inductive approach will first be used to gain direct information from study participants, without imposing preconceived categories or theoretical perspectives, and to ensure that knowledge generated from the content analysis is based on the participants’ unique perspectives [27]. After themes have been derived through inductive content analysis, a deductive approach will be used as the final step to frame and structure the findings [26]. Therefore, the themes generated through inductive content analysis will be delineated and mapped in accordance with Chaudoir’s multilevel framework for the assessment of factors affecting the implementation of health innovations [28].

## Results

Patient recruitment began in January 2019 at PMCC, UHN, Toronto. The study is currently in progress, and a total of 76 participants have been recruited as of February 24, 2020 (39 in the intervention group and 37 in the control group). The final analysis is expected to be completed by the winter of 2021. This study will be among the first to substantiate the implementation of remote titration facilitated by telemonitoring and its impact on patient health outcomes.

## Discussion

### Principal Results

This study aims to determine how the combination of remote titration and telemonitoring affects GDMT optimization compared with the standard of care. Specifically, the objectives of this study are to assess the effectiveness and implementation of remote titration facilitated by telemonitoring. Telemonitoring is a potential component in the management of HF that allows patients to remotely provide reliable and real-time physiological data for clinical decision support. Studies have demonstrated that the use of telemonitoring in the HF population is associated with a reduction in hospitalizations and readmissions and improved mortality [29-32]. Patient and clinician perceptions are positive, and telemonitoring is viewed as a useful and efficacious tool that can be used to promote positive outcomes in the HF population [33-35].

Despite this, only a few trials have attempted to use telemonitoring for the purpose of remote titration of HF medication. A study conducted by D’Onofrio et al [36] and Palmisano et al [37] assessed the effectiveness of a structured program for BBL titration and found that remote titration allowed 76% of patients in the intervention group to achieve target doses compared with only 38% of patients in the control group. Similarly, Spaeder et al [38] also performed a study that focused on rapid titration of the BBL carvedilol and compared in-office titration with a combined in-office and remote titration model. The study found no statistical difference in the proportion of patients who reached the target doses. However, the time frame required to reach the final dose was significantly shorter in the intervention group (mean 33.6, SD 16.6) than in the control group (mean 63.7, SD 20.2).

Of note, a few other studies that have attempted to perform remote medication titration did not contain any telemonitoring components, such as a smartphone-based app or a web platform. Instead, patients periodically called or were contacted by a clinician and relayed their measurements and symptom data over the phone. Two such trials by Steckler et al [39] and Moyer-Knox et al [40] assessed BBL titration over the phone. Steckler et al [39] found that the proportion of patients receiving BBLs at any dose increased from 61% at baseline to 97% after optimization, and the proportion of patients receiving target BBL doses increased from 12.5% at baseline to 40.6% after optimization. Moyer-Knox et al [40] found that 96% of patients reached therapeutic doses (6.25 mg twice daily) and 71% of patients reached target doses of 25 mg twice daily within approximately 8 weeks.

These trials provided preliminary evidence demonstrating that remote titration (with or without the aid of telemonitoring) of BBLs can be successful and results in a higher proportion of patients reaching target doses within shorter time frames.

### Strengths and Limitations of the Study

Unlike previous studies that focused solely on the titration of BBLs, a strength of this study is that it encompasses the titration of full triple therapy for patients with HF. In addition, the mixed methods design of this study will allow triangulation of data from quantitative and qualitative assessments, thereby enhancing data validity. Methodological triangulation enables the validation of findings through the collection of data from multiple sources and via different methods. Specifically, data from interviews with clinicians and patients will be used to complement, confirm, and explain the results of the quantitative study. Furthermore, the previously existing evidence regarding remote titration and the currently available data on the effectiveness of Medly telemonitoring for patients with HF [22,23,33,41] make it possible to adopt an effectiveness-implementation hybrid design [17]. Therefore, implementation-related questions can be explored much earlier than could be achieved in separate sequential intervention and implementation study designs [17].

A limitation of this study is its single-center nature and the availability of dedicated specialized staff to support the intervention. The patient population enrolled in this study was recruited from a single specialized heart function clinic. The PMCC Heart Function Clinic has implemented telemonitoring as a standard of care. The Medly Program was launched at the clinic in 2016, and cardiologists are familiar with monitoring patients through Medly. The familiarity of the clinicians involved in this study with telemonitoring as well as the existing processes for alert management and communication of information obtained through the Medly system may contribute to the mitigation of challenges that could otherwise be encountered. Furthermore, the intervention is supported by a dedicated nurse coordinator. As access to multidisciplinary HF services varies between clinics, additional staffing limits the potential generalizability and external validity of the study. Finally, as our study investigates changes in the process of care, blinding could not be applied to clinicians. However, patient randomization is performed using sealed opaque envelopes containing randomly generated treatment allocations. Patients in the intervention group follow a structured predetermined remote titration schedule, whereas patients in the control group continue to be treated as per the standard of care by their respective cardiologists. Thus, the lack of clinician blinding is not expected to have an impact on the outcomes of the study.

### Significance of the Research

The significant gap that still exists in adherence to guideline-recommended evidence-based therapies for HF emphasizes the need for novel approaches to the problem of medication titration. An intervention that can successfully promote optimal GDMT use in clinical practice may substantially improve clinical outcomes in patients and reduce the burden of HF on the health care system as a whole. Although information and research on remote titration of HF medication are somewhat limited, the results of previously conducted studies have been fairly positive, pointing to a favorable impact on titration rates and timelines. This study will be among the first to explore whether remote titration facilitated by telemonitoring may be able to promote optimal GDMT use. In addition, it will be the first study to provide insight on the implementation process as well as the perception of the intervention by both clinicians and patients.

## Abbreviations

ACEI: angiotensin-converting enzyme inhibitor
ARB: angiotensin receptor blocker
ARNI: angiotensin receptor-neprilysin inhibitor
BBL: beta blockers
BNP: brain natriuretic peptide
CPET: cardiopulmonary exercise testing
ECG: electrocardiogram
EPR: electronic patient record
GDMT: guideline- directed medical therapy
HF: heart failure
LVEF: left ventricular ejection fraction
MRA: mineralocorticoid receptor antagonist
NYHA: New York Heart Association
PMCC: Peter Munk Cardiac Centre
RCT: randomized controlled trial
UHN: University Health Network
